# A Workflow for Meaningful Interpretation of Classification Results from Handheld Ambient Mass Spectrometry Analysis Probes

**DOI:** 10.3390/ijms25063491

**Published:** 2024-03-20

**Authors:** Alexa Fiorante, Lan Anna Ye, Alessandra Tata, Taira Kiyota, Michael Woolman, Francis Talbot, Yasamine Farahmand, Darah Vlaminck, Lauren Katz, Andrea Massaro, Howard Ginsberg, Ahmed Aman, Arash Zarrine-Afsar

**Affiliations:** 1 Princess Margaret Cancer Centre, University Health Network, 101 College Street, Toronto, ON M5G 1L7, Canada; a.fiorante@mail.utoronto.ca (A.F.); lananna.ye@uhn.ca (L.A.Y.); michael.woolman@uhn.ca (M.W.); francis.talbot@uhn.ca (F.T.); yasi.farahmand@mail.utoronto.ca (Y.F.); darah.vlaminck@mail.utoronto.ca (D.V.); lauren.kaufman@mail.utoronto.ca (L.K.); 2 Department of Medical Biophysics, University of Toronto, 101 College Street, Toronto, ON M5G 1L7, Canada; 3 Istituto Zooprofilattico Sperimentale Delle Venezie, Viale Fiume, 78, 36100 Vicenza, Italy; atata@izsvenezie.it (A.T.); amassaro@izsvenezie.it (A.M.); 4 Ontario Institute for Cancer Research (OICR), 661 University Ave Suite 510, Toronto, ON M5G 0A3, Canada; taira.kiyota@oicr.on.ca (T.K.); aaman@oicr.on.ca (A.A.); 5 Department of Surgery, University of Toronto, 149 College Street, Toronto, ON M5T 1P5, Canada; howard.ginsberg@unityhealth.to; 6 Keenan Research Center for Biomedical Science & the Li Ka Shing Knowledge Institute, St. Michael’s Hospital, 30 Bond Street, Toronto, ON M5B 1W8, Canada; 7 Department of Laboratory Medicine and Pathobiology, University of Toronto, 1 King’s College Circle, Toronto, ON M5S 1A8, Canada; 8 Leslie Dan Faculty of Pharmacy, University of Toronto, 144 College St, Toronto, ON M5S 3M2, Canada

**Keywords:** ambient mass spectrometry, picosecond infrared laser mass spectrometry, rapid pathology, lipidomics

## Abstract

While untargeted analysis of biological tissues with ambient mass spectrometry analysis probes has been widely reported in the literature, there are currently no guidelines to standardize the workflows for the experimental design, creation, and validation of molecular models that are utilized in these methods to perform class predictions. By drawing parallels with hurdles that are faced in the field of food fraud detection with untargeted mass spectrometry, we provide a stepwise workflow for the creation, refinement, evaluation, and assessment of the robustness of molecular models, aimed at meaningful interpretation of mass spectrometry-based tissue classification results. We propose strategies to obtain a sufficient number of samples for the creation of molecular models and discuss the potential overfitting of data, emphasizing both the need for model validation using an independent cohort of test samples, as well as the use of a fully characterized feature-based approach that verifies the biological relevance of the features that are used to avoid false discoveries. We additionally highlight the need to treat molecular models as “dynamic” and “living” entities and to further refine them as new knowledge concerning disease pathways and classifier feature noise becomes apparent in large(r) population studies. Where appropriate, we have provided a discussion of the challenges that we faced in our development of a 10 s cancer classification method using picosecond infrared laser mass spectrometry (PIRL-MS) to facilitate clinical decision-making at the bedside.

## 1. Introduction

The past decade has seen the promising development of various hand-held mass spectrometry analysis probes [1]. These probes combine a direct (from a solid specimen) sampling or “desorption” method under ambient conditions with an appropriate ionization interface for real-time analysis of a specimen’s molecular content by way of profiling its heterogeneity in the mass-to-charge (*m*/*z*) space. Coupled with (a) multivariate statistical analysis method(s), these hand-held probes strive to shorten the timescale of clinical diagnosis, add more “molecular depth” to conventional diagnosis workflows, or assess the suitability or the adequacy of an acquired specimen for conventional histopathological analysis [1,2,3,4,5,6,7,8,9,10,11,12,13,14,15]. Most notably, many such probes can utilize ex vivo or in vivo biological tissues without the need for specimen processing before analysis. The hardware configurations for such probes are reviewed extensively elsewhere [1], alongside visions for their utility in future clinical diagnosis [4,16,17,18], especially during surgery [2,5], and need not be re-reviewed here.

To facilitate the clinical translation of hand-held mass spectrometry analysis tools from bench to bedside, several metrics related to analytical performance and regulatory compliance must be met. Our group has recently reviewed these topics in two separate publications, putting forward recommendations for a suitable design of experiments to meet these requirements [13,19]. The first publication [13], through drawing parallels with the validation guidelines for United States (US) Pharmacopeial untargeted analyses [20] and related laboratory-developed tests [21], as well as FDA approvals of two untargeted matrix-assisted laser desorption ionization mass spectrometry (MALDI-MS)-based assays for infectious bacterial agent identification [22,23], proposed harmonized guidelines for the validation of ambient mass spectrometry methods in clinical decision-making, which are consistent with sentiments summarized elsewhere [2,16,17]. To complement this proposal, the second review paper from our group [19] utilized lessons from failures of laboratory-validated proteomic biomarkers at the implementation stage [24], sought to further refine said validation workflow in a manner that recognizes, and thus addresses by its design, post-validation pitfalls associated with “late-stage” implementation issues “in transition” (en route) to clinical translation. These recommendations ranged from clearly defining the clinical added value before embarking on time-consuming validation to recruiting specimens for training and validation datasets that are consistent with the anticipated heterogeneity at the population level at the laboratory validation stage. These recommendations are aimed at avoiding failure at the implementation stage, which is much more “costly” for the research team involved than any “pre-transition” disappointments [19]. Additionally, a related review paper from our group critically evaluated the choice of a suitable biological material in ambient mass spectrometry studies aimed at cancer research [25], highlighting the importance of early access to human patient tissue. This sentiment may not be palatable to those who believe that animal models are best suited for early-stage or proof-of-concept studies due to their ease of access and the need for conservation of precious banked specimens, accessing them only upon validation of feasibility using “lower” disease models. However, the results presented therein [25], both from our laboratory [11,26] and gathered from the literature, support a significant discordance between the ambient mass spectrometry profiles [27] of primary human tissue and those of immortalized cell line, as well as murine xenografts (consistent with genomic analyses [28]). This positioned primary human tissue or patient-derived xenografts as the most suitable for being studied in the earliest stages of marker discovery [25]. Here, the strengths of murine models lie within one’s ability to use genetic modifications to orthogonally validate the markers that are discovered through direct human tissue studies [25]. For example, gene knockout or knockdowns can be used to verify the involvement of a metabolic pathway that is identified through human tissue studies. We would like to add that if the ambient hand-held analysis probe in question is compatible with analysis of formalin-fixed paraffin-embedded tissue (FFPE), initial studies could utilize FFPE specimens that are much more abundant in tissue banks that frozen specimens. However, a key point to remember is that the concordance of FFPE and fresh tissue signatures must first be established to rationalize the investment made in creating profile libraries with embedded tissue for the rapid classification of fresh surgical specimens.

In the quest to successfully design a clinically relevant ambient mass spectrometry analysis project, the publications described above cover a breadth of relevant material. However, they fail to ensure meaningful interpretation of ambient mass spectrometry results, given the need for statistical rigor in both the experimental design and interpretation of results phases. To make matters more complicated, untargeted mass spectrometry datasets often possess a large within-group variation, and this affects the rigor with which between-group discriminations can be made [29]. Here, the cornerstone in one’s ability to assess the utility (and/or reproducibility or performance) of any newly developed method is a multicentre study, wherein users with a pre-defined technical skillset [13] follow a written knowledge translation (KT) document to gain competence in utilizing the method and interpreting the results. The envisioned KT package must be comprehensive and alert users to potential pitfalls and relevant mitigation strategies in each step of the experimental design and execution, and it must pay special attention to “rigor” in data analysis (for the validity of the conclusion that is drawn), in addition to the basics, such as how to operate the associated hardware. As such, at the heart of a successful KT package must lie a transparent discussion of the known shortcomings of the method, the sources of variance (or instability) in the hardware and/or in the generated data, alongside complications that prevent a robust interpretation of results, including improper experimental design. Building on previous comprehensive reviews described above that discuss validation strategies [13], the pitfalls of ambient MS analysis with hand-held sampling sources [19], and a choice of experimental tissue types that favors the use of human tissue in the early stages of design and implementation [25], we propose a workflow for meaningful interpretation of ambient mass spectrometry analysis results. We used the rapid analysis of clinical specimens with picosecond infrared laser mass spectrometry (PIRL-MS) as an example, emphasizing the experimental design, data analysis, and interpretation of results. As PIRL-MS is not a current commercial product, we have refrained from discussing elements that detail the operation of the associated hardware, which is currently vendor-specific. This manuscript, nevertheless, is based on a currently implemented device in our laboratory in conjunction with a PIRL-V laser unit from Light Matter Interaction (Etobicoke, Ontario, Canada) and a Xevo G2-XS time-of-flight mass spectrometer (Waters, Milford, MA, USA), as well as the Abstract Model Builder software (AMX, Waters Research Centre, Budapest, Hungary, version 1.0.1360.0).

Below, we first provide a summary of potential pitfalls in the analysis workflow and sources of variance in each step of the analysis workflow. We then recap previous knowledge and accordingly discuss mitigation strategies, working towards a pictorial diagram that summarizes the envisioned workflow. The manuscript will be emphasizing the necessary aspects for a successful experimental design and robust interpretation of results. We intend this workflow to be a “living document” and be updated as new sources of variance and shortcomings come to light, accommodating the unknown unknowns of the future. Where appropriate, we have proposed practical approaches to draw users’ attention to known unknowns that could complicate the analysis workflow. Given that many current users of hand-held mass spectrometry analysis probes (including our group) may lack a deep understanding of statistics, we have strived to put forward “practical” guidelines to verify the sources of analysis failure. These include, but are not limited to, a lack of sufficient specimen numbers and overfitting of the data, which is a common occurrence in multivariate datasets that contain many more variables (or features) than the independent samples that are used to create them [29,30]. These practical guidelines are not intended to replace the need for a rational design of statistically robust experiments using multivariate methods [31,32]. We do encourage the involvement of statisticians in all stages of the experimental design, calculations of a suitable study size, and selection of appropriate cohorts given the anticipated levels of inter- vs. intra-specimen variations. These guidelines are meant to highlight the usefulness of simple control studies such as scrambling the data to validate the significance of multivariate observations that have been made. These simple, yet highly informative, controls are currently lacking in many reported applications, especially those that are based on teachings of molecular models constructed from only a few independent specimens, without orthogonal assessment with an independent validation cohort. In our discussion below, where appropriate, we have adapted from parallel studies utilizing multivariate analysis of mass spectrometry fingerprints in food fraud detection [29]. While the proposed guidelines [29] are not currently officially recognized by accrediting bodies, they constitute a close match with challenges that are faced in mass spectrometry fingerprinting of clinical specimens, which is the focus of this review paper.

## 2. Experimental Design

### 2.1. Prospective, Retrospective Approaches and Appropriate Patient Inclusion/Exclusion Criteria

The first step involves establishing appropriate patient inclusion and/or exclusion criteria, as well as a comprehensive list of clinical metadata including co-morbidities or additional factors that are known to affect the mass spectra. Here, the users are faced with two key questions that must be immediately answered, as the responses to these questions are required to seek the necessary institutional authorizations before the study can begin. These questions are as follows: (1) how many independent specimens must be included in the study design for observations to be statistically valid, and (2) will a retrospective (banked tissue) study be sufficient in terms of the clinical metadata that must be taken into consideration for a meaningful interpretation of results, or must a prospective tissue collection effort that allows for patient interviews to achieve a parallel collection of clinical data be followed? For example, a repository that banks lung cancer specimens may have access to patient records, but such records may not contain information on whether the patient was a smoker or not. Therefore, should tobacco use be deemed necessary metadata, a prospective study is likely more suitable. In the quest to implement a prospective tissue collection process, however, another complication quickly arises: is the incidence rate of the indication in question (following our analogy, lung cancer) at the providing healthcare centre or institution sufficient to allow the necessary number of independent specimens to be collected during a reasonably short amount of [study] time? Note that we have not yet defined what that number is and are faced with this additional complication. With respect to the suitability of sample numbers, it must be emphasized that the variance in multi-feature datasets is a complex function of the relative ratio of inter- vs. intra-group variability. Here, in principle, there are multivariate power calculations to be consulted in rationally designing the study size [33,34,35,36,37]. However, how would one approach modeling these calculations in the absence of any previous experimental data to shed light on the extent of such variance? To make the matter more complicated, the study design likely involves attempts to discriminate between at least two indications (following our analogy above, e.g., lung cancer from normal lung tissue or differentiating between different histological types/classes of lung cancer). Here, an additional layer of inter- versus intra-class variance comes into play, influenced by the presence of further molecularly distinct subclasses contributing to spectral heterogeneity. To address this question, a fundamental point must be revisited regarding the specimen’s molecular heterogeneity and possible subclasses. The user could design the experiment to reduce the intra-class variance and accentuate any anticipated inter-class differences by utilizing a smaller cohort of carefully selected patients, who are positive for the indication in question but negative for any other competing (in terms of influencing mass spectra) indication compared to the control group. Alternatively, a very large study cohort could be used to ensure that all other competing factors such as co-morbidities (hypothetical or not) and their influences on mass spectral heterogeneity immediately become a washout. This, however, may result in a significant demand for normal tissue samples, which are often more difficult to obtain. It must be emphasized that in spatially resolved studies such as mass spectrometry imaging, the influence of tissue heterogeneity is much more easily captured and addressed, as opposed to in MS profiling with hand-held probes. These methods simply rely on a “blind”’ interrogation of a desired spot on a resected or in situ specimen and are often not guided to “information-rich” areas of the specimen. This is a limitation that warrants capturing as much of the heterogeneity (in terms of variance in signal) as possible in molecular models to optimize their performance. Having said this, however, we strongly recommend utilizing spatial information in models. For example, one can envision an improvement in the model quality by including stromal signatures or infiltration biomarkers (as two examples) obtained via spatially resolved mass spectrometry imaging to achieve successful recognition of hand-held point probe data. An example of this is described in a previous study by our group, wherein we attempted to classify hand-held probe data with spatially resolved PIRL-MS models [38].

### 2.2. Suitable Study Size and Data Acquisition Strategy

In addition to the spatial heterogeneity discussed above, there are other factors that influence data heterogeneity. Here, while higher-order data analysis methods do exist to disentangle the influence of other variances such as those resulting from co-morbidities and other competing factors on mass spectral profiles, parallel studies in untargeted medical mass spectrometry [39] and food sciences [40] strongly support the inclusion of representative specimens from the expected diverse cohort, even if initially, the models created do not offer the robustness that is needed for long-term future use (they can be updated periodically and approved for subsequent use [40]). Therefore, gathering as much background information as possible regarding the extent of the anticipated mass spectrometry variance across the population through clinician interviews, reviewing disease pathway information (if known), or mining the literature for parallel molecular studies (proteomic, transcriptomic, etc.) may prove helpful. Here, it must be noted that there is no one-to-one correspondence between genomic and phenomic datasets per se [41], and some types of genomic variance that arise due to clonal evolution [42] may be washed out at the metabolite level [11]. Additionally, many lifestyle and dietary factors [43,44], including taking certain hormonal supplements or oral contraceptives [45], have metabolic impacts [19]. These, however, are likely best addressed at later stages of prospective tissue studies rather than retrospective banked tissue efforts, as such metadata, e.g., oral contraceptive use, may not be well documented in the consented-to clinical metadata that accompany patient records for banked specimens. Due to the complications discussed above, at the heart of which is a lack of an a priori estimate of the inter- versus intra-class variance, we recommend an empirical approach based on previously available PIRL-MS results that compares the statistical significance (using quantitative cluster overlap) across two extreme datasets of (1) homogeneous molecularly distinct samples (small intra-class variance) and (2) heterogeneous molecularly similar samples (small inter-class variance) that have been published previously [19]. We recommend beginning the study with at least 50 retrospectively acquired specimens for creating the very first multivariate molecular model. To determine this number, we used human medulloblastoma cancers comprised of four morphometrically identical yet molecularly distinct classes, wherein we required ~20–25 independent specimens per class to produce distinct data clusters [11]. As the presence of additional heterogeneity could further reduce cluster compactness, necessitating additional specimens per class, we applied a factor of two to this empirical observation as a safety margin for error. As such, the recommended 50 specimens per class is only being considered a starting point and is to be verified and/or adjusted, as detailed below. It must be emphasized that less stringent specimen numbers per class could be considered in cases where the molecular variance between classes is much larger than the intra-class variance (see below). Additionally, some biospecimen repositories only utilize synoptic pathology reports (that in nature are succinct) to annotate the pathology indications. As such, there could be a discordance between the banked specimen aliquot and the specimen aliquot that is subjected to clinical diagnosis, results of which are included in the pathology report. We thus strongly recommend engaging a pathologist to verify the presence of the expected pathology in the actual specimen that is dispensed and subjected to ambient mass spectrometry analysis.

To simultaneously capture both inter- and intra-specimen heterogeneities, we recommend performing multiple samplings across the surface of each independent specimen, as well as documenting the location of each sampling event for post-sampling analysis of the specimen’s gross histopathology [11,12,26]. Therefore, specimens with a sufficient surface area are generally highly desirable. Having multiple spectral data points across a specimen’s surface will allow for the calculation of the extent of spatially invariant or concordant classification (or precision) for the method [11], which is quite desirable to demonstrate. Ideally, the MS-based pathology predictions should be independent of the location of sampling on the specimen (unlike some genomic methods that are overly sensitive to clonal evolution [42]). The mass spectral datasets can be subjected to multivariate modeling, both per sampling event and per specimen (by way of randomly selecting a representative sampling event’s data). We are ambivalent concerning averaging the spectral information across multiple sampling events, because in future clinical implementation, this will rarely be relevant. A successful hand-held MS analysis probe must deliver concordant pathology assessments irrespective of the site of sampling across the specimen’s surface, and averaging the spectra across areas of gross histopathologic variance may not translate to meaningful results. In addition, acquiring the data and treating each sampling event as a discrete data point will allow for tracing of the MS data to areas of tissue heterogeneity as carried out previously with the aim of a more reproducible interpretation of model failures [11]. Establishing fiducial markers to be able to trace each sampling event (or data point) in the coordinate of the specimen by either using unique sampling patterns or video recording the data collection will be key in being able to correlate sampling events to specimens’ gross histology post-analysis, as carried out previously [11]. To ensure that the inclusion of dependent (from the same specimen) data points in a molecular model does not cause complications in validation, caution must be exercised, as detailed below. In summary, we recognize that some of our stringent recommendations regarding the ideal specimen numbers and gathering suitable clinical metadata and heterogeneity information, extrapolated logically from the degree of variance that we have seen in closely related solid tumours in our experiments, may simply not be feasible for rare cancers. As mentioned above, we do encourage engagement of statisticians in the early phase of the study design to explore alternate analytic strategies that are suitable for small studies.

## 3. Creation and Validation of Mass Spectrometry Molecular Models Using Intelligent Data Analysis Tools

### 3.1. Data Analysis Approaches and Preprocessing of Data

Multivariate modeling remains the most utilized method of data analysis in ambient mass spectrometry [3,7,10,11]. A variety of modeling tools are employed that often require “preprocessed” mass spectra. The collapse of the spectral information content into data bins of a lower resolution is a common practice for time-of-flight instrument data that otherwise require real-time correction of mass drift. This will also ensure that the rapid processing of data can take place in the absence of mathematical treatments that are required for centroid peak fitting and high-resolution work. Here, an understanding of an effective “resolving power”, needed to distinguish appropriate classes under study, must first be established. In PIRL-MS, “soft ionization” largely uncovers relatively sparse native charges, resulting in a suitable balance between spectral complexity and relative simplicity that can accommodate low-resolution analyzers. While many hand-held MS analysis probes have been coupled to high-resolution mass analyzers, a bin window of 100 mDa has generally been sufficient for PIRL-MS [11,12,26,46]. Higher-resolution mass analyzers may confer an added benefit in the case of targeted analysis using a few *m*/*z* peaks, especially if there is a concern regarding spectral overlap. However, spectral “simplicity” could also morph into an oxymoron to contend with. “Poor-quality” mass spectra can also be “simple”, in that they may lack sufficient information content across many bins. Therefore, including them in multivariate analyses may result in a “garbage in, garbage out” type of assessment. This warrants careful implementation of data quality “checkpoints”, after first establishing metrics for “good-quality” spectra using characteristics like the presence of expected *m*/*z* peaks, overall ion count, and/or signal intensity thresholding, among others. We can then exclude “poor-quality” data from the analysis by applying the same criteria equally to all specimens that are utilized in the study. It is, however, conceivable that some tissue types may intrinsically yield lower-intensity spectra than others. It is thus important to revisit the suitability of the data quality criteria for each experimental design. Likewise, normalization of the mass spectral data to total ion intensity (while useful in most cases to shield against noise) may create a bias, leading to false classification of aberrant poor-quality spectra from a specimen type with inherently strong spectral intensity being recognized by the model as a member of the other class with intrinsically poor signal levels. Here, other normalization strategies such as to use a non-zero median [47] or median-fold change log transformation [48] have also been reported, alongside normalization with TIC and/or with background subtraction, by other groups [49,50]. As such, we recommend that investigators establish metrics for “good-quality” data prior to any modeling effort and subsequently perform a post-modeling inspection of all misclassified or failed data points or those that fall between expected classes in the multivariate model for both data quality and the presence of aberrant histology/clinical metrics potentially not captured in the model. Here, “pruning” or cleaning up the molecular model on the grounds of outlier data that do not conform to expected clusters (or cluster ends) does not constitute a sound scientific practice. All data points that are deemed to be outliers should be further investigated to rationalize why they failed to cluster with the expected group. This was recently reported in the case of a badly damaged brain cancer specimen suffering from electrocautery artifacts that spectrally did not resemble any of the competing classes that were present in the multivariate model [11]. Here, it must be emphasized that the analysis methods that allow for determination of a probability value for the assignment of data points to an accepted class provide an opportunity to improve the diagnostic accuracy of predictions based on thresholding the class assignment probabilities without manipulating or pruning the model itself. This is of importance in clinical analysis because the burden (or clinical consequence) of a false prediction is often considerably higher than that of an unclassifiable (outlier, no prediction) assignment, based on which no clinical decision will be made. A similar concern for the “asymmetry of [consequences] of error” in class prediction has also been discussed in the context of adulterated food detection with untargeted mass spectrometry [29]. Heightened levels of stringency in the probabilistic acceptance of class assignments, however, can lead to a reduced duty cycle (and limit the overall utility of the method), with the model simply not recognizing a significant number of validation or test spectra. A practical balance in what constitutes an acceptable probabilistic prediction stringency must be reached. An example of the utility of probability thresholding has been demonstrated by our group previously [11]. In that work, increasing the prediction probability threshold without altering the model resulted in a reduction in misclassified data and improvement in class prediction accuracy [11]. This approach was motivated by the fact that no clinical decision is made for unclassifiable data points. Therefore, their exclusion from the classification statistics may be warranted [11]. While we recognize that such an approach may be controversial to some readers due to the underlying assumptions that are made for probabilistic decision-making (i.e., excluding outliers or unclassifiable data from our accuracy calculations), we believe that it has merits in current implementations of untargeted mass spectrometry in cancer research. Here, repeated sampling of the specimen can be performed until classifiable prediction results are achieved to satisfy a user-defined threshold of prediction probability. Under such stringent conditions, the resulting classification is bound to be highly accurate, and this is preferable for implementation. Here, the correct diagnosis could potentially be delivered upon measuring the first classifiable data point, and with the goal of including high-quality data in prediction algorithms, the duty cycle will be further optimized. The concept of probabilistic decision-making in clinical diagnosis and treatment planning has existed and been practiced, debated, and improved for a long time [51,52,53]. The concept stems from the fact that in certain clinical practices, no absolute diagnosis with 100% certainty is possible to reach in order to motivate a concrete decision. Therefore, a threshold must be established below which the accuracy of a diagnosis is called into question (to be retested), and over which the diagnosis is robust and thus accepted. When a molecular model for mass spectrometry fingerprints can deliver a diagnosis (i.e., class assignment) along with a probability value for said assignment (i.e., diagnosis), a parallel scenario is created that allows for easy utilization of the “threshold [decision making] model” described previously [51,52,53]. Here, diagnoses associated with a lower-than-threshold probability should be retested (e.g., when unclassifiable data have been obtained), and those over the said threshold should be taken as accurate. We believe that by adapting from the probabilistic decision-making principles using the threshold model [51,52,53], we can improve the diagnostic accuracy of mass spectrometry fingerprinting.

Concerning the criteria for “good-quality” data, in our current implementation of PIRL-MS, we have empirically determined that a minimal signal duration of 3 s (three 1 s time-of-flight scans) and an intensity of >10^3^ are considered suitable for soft tissue cancers, while sampling over 10 s did not show additional improvements in spectral quality or classifiability, at least in the cases that we examined [11]. Currently, we collect and log every attempt of spectral collection to obtain an honest calculation of the method’s duty cycle. However, in our experimental design, we often inform the PIRL-MS probe operator of the data quality in real time by visual inspection of live spectra by an experienced user, so that the probe operator can adjust the collection geometry in real time (e.g., move to a different spot on the specimen or clean the laser fiber) if needed. We have adopted this strategy to maximize the number of good-quality obtainable data from a given sample. While this practice is very useful for ensuring that several good-quality spectra are recorded from small or scarce specimens, the process must be automated to inform end-users of the suitable data quality in the absence of visual inspection of live spectra. This would allow “good quality” data to be collected even in the absence of an expert mass spectrometrist visually evaluating the real-time spectra, guiding the user to collect additional data points, or end the data collection to save time. This is applicable to both (1) modeling, where a significant number of good-quality data must be recorded, as well as to (2) validation, where robust analysis depends on having acquired a desired number of good-quality spectra for prediction using the model. In this context, the spectral repeatability could be judged based on the coefficient of variance (CV) without user intervention, as suggested in [54], as well as by a cosine of similarity approach [15,55] and its variants. It is imperative to note that the instrument “health” must be checked before data collection. In our laboratory, we routinely use mouse organs (easily accessible and relatively homogeneous) as Quality Control (QC) and proceed to data collection provided that classifiable data points (against a previously established molecular model of mouse organs) are obtained. Therefore, achieving an expected classification can also be used as a metric for “good-quality” spectra. This may constitute a more practical approach to determining the suitability of data for untargeted analysis compared to the spectral similarity approaches mentioned above, as the relationship between the spectral similarity indices (such as the CV threshold cut-off) and “classifiability” is not yet understood. Having said this, nevertheless, elucidating the relationship between spectral similarity and “classifiability” (using the desired molecular model) may prove beneficial, as it may lead to data-independent rulesets being used in data QC before the mass spectra are sent to the model for classification. Here, mining the poor-quality spectra for developing the above-mentioned ruleset is recommended. In summary, opportunities for the development of appropriate data QC strategies, such as by way of aliquoted and carefully stored tissue homogenates, exist. Overall, for modeling purposes, it will be desirable to only include repeatable spectra in classifiers with duplicate non-repeatable spectra of the same sample that are subjected to re-analysis to establish whether they correspond to real biological variables in the specimen, such as heterogeneity.

### 3.2. Data Analysis: First Steps and Approach Planning Considerations

Once the collection of data has been completed, data analysis begins. A suitable first step is to explore the potential for class distinctions using the acquired data. Here, an unsupervised method such as principal component analysis (PCA) will be useful to glean insights into the relationship between classes and their spectra. This method provides good “visuals” of the potential for class distinctions, highlighting patterns in data groupings and the relationship between supervised class annotations and unsupervised data-driven classes to be explored next. The subsequent steps involve revisiting the data using supervised methods. These methods often accentuate inter-class distances, wherein the potential for overfitting must be carefully monitored, as detailed below. While a collection of very different statistical modeling methods, including multivariate approaches coupled to linear discriminant analysis (e.g., principal component analysis linear discriminant analysis or PCA-LDA), least absolute shrinkage and selection operator (LASSO), as well as more advanced learning methods such as random forest (RF), support vector machine (SVM), and neuronal networks (NN), have been used in ambient mass spectrometry analysis [1,2,3,5,10,11,26,31,39,47,56,57,58,59,60,61,62,63,64,65,66], the current implementation of PIRL-MS used multivariate modeling with supervised PCA-LDA [31,32]. This method is computationally not costly, but it requires careful calculation of the number of components and dimensions required for the analysis to avoid overfitting [67]. To calculate a suitable number of components, “Scree plots” [68] can be used. These plots show the extent of variance that is used for each of the utilized PCA components. However, our current workflow uses an empirically determined maximum principal component number approximating one-fifth (20%) of the total number of data points, with the number of linear discriminant dimensions being the total number of classes minus one [11,12,46]. The maximum number of dimensions in a PCA test is often equal to the number of variables that is used [68]. The rationale for the empirical maximum number above is the average number of true resolvable features in our mass spectra and may need to be adjusted or rationally evaluated in parallel studies and adaptations, as discussed [68]. It is no secret that deviations from these benchmarks may lead to overfitting of the data in the model. This is further discussed in Section 3.3. Such an estimate constitutes the maximum possible PCA components. In our laboratory, we routinely use PCA in combination with LDA, and often, tens of components are sufficient to provide robust analysis without overfitting, especially if only a few classes (to be separated by supervised LDA) are present. However, in the absence of using “Scree plots”, how do we ascertain that such empirically determined suitable PCA maximum component values themselves do not result in data overfitting in the first place? To date, we have validated our multivariate models by demonstrating their ability to predict the “ground truth” pathologies for an independent set of test specimens, processed using similar parameters overlayed onto the model. We have additionally used Mahalanobis [69] distance calculations to produce a probability value for said class prediction by taking into consideration the distance between the accepted (predicted) class and all other rejected classes [11,12] (this was carried out using AMX [70]). There are, however, points of caution to be considered. Most importantly, as additional data are added to the model, the cluster density or compactness inevitably becomes influenced by the number of specimens that are included in each cluster, as each specimen contributes ever so slightly to the cluster variance [71]. In addition, day-to-day instrumental variation could be at play. As such, we recommend the use of balanced sample numbers for each class, resulting in equal data points across each cluster, collected from similarly sized independent specimens. We additionally suggest the study data to be collected over a series of days so that both day-to-day instrumental, as well as the specimen’s natural biological, variances are captured by the model. Recognition of a class based on the Mahalanobis distance in overlap tests [71] requires a strict definition of a standard deviation from a cluster boundary and/or centre of mass. Thus, cluster shape(s) may need to be taken into consideration before a suitable scoring algorithm can be defined, especially if distances are calculated based on deviations from the centre of mass of the cluster, as opposed to its boundaries for example. Here, while the use of balanced sample numbers may additionally help, it is often the intra-class variance that defines the cluster shape. Therefore, two classes with highly different intra-class variance may result in divergent cluster shapes, and this could be further confounding, as ideally, a model should capture as much of the expected variance in each class as possible, including specimens collected at various time frames. This, however, may allow a large model cluster to correctly classify an overlapping yet much more compact cluster. However, the inverse may not be true, unless a very large standard deviation is chosen for the Mahalanobis measurements, which may elevate the risk of false discovery (i.e., incorrect class prediction) during model validation with the test cohort.

**Figure 1:**
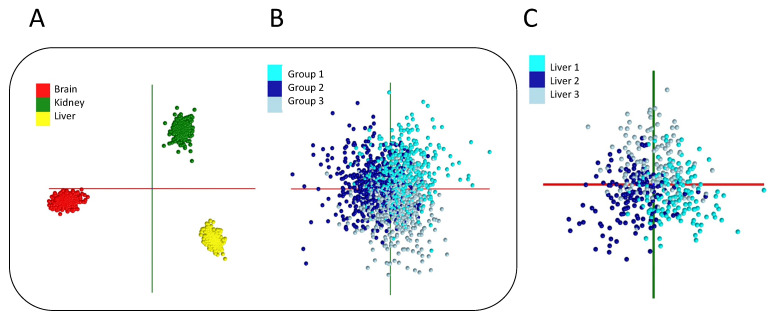
Model validation using mixed class permutations. (**A**) PCA-LDA score plot of a model created from 10 independent mouse kidney (442, 10-s PIRL-MS sampling events), 10 independent mouse brain (446, 10-s PIRL-MS sampling events), and 10 independent mouse liver (409, 10-s PIRL-MS sampling events) samples. The data for this model were acquired as described previously using the reported hardware [46] and processed using 1/5th (20%) of the number of data points as the maximum number of PCA components (this was equal to 258 for 100% data usage). As shown here, a separate grouping of data points between classes is seen. (**B**) The PCA-LDA scores plot of a permuted model with mixed classes, wherein each group contains an equal representation of data from all other classes. This model does not show any separation of data, suggesting that the separation seen in the class data in panel (**A**) (with true class annotations) is significant. This model was processed using the same parameters that were used for panel (**A**). This is key. Panel (**C**) shows the PCA-LDA score plot of 409 data points (sampling events) from 10 independent mouse livers, analyzed in panels (**A**,**B**) and divided equally between three repetition classes, each containing 132, 138, and 139 events, respectively. This model serves as a control and shows a similar degree of mixing as what was shown for the permuted false class model of panel (**A**). Figure 2 shows the cross-validation statistics of these models (at 10% sample use intervals) from the 20% leave-out test and suggests 97.84%, 44%, and 51% for panel (**A**–**C**) models, respectively, at 100% data usage. For Figure (**A**,**B**) models, the maximum number of PCA components was 258. Reducing this to only 25 also resulted in cross-validation of 99.69% for (**A**), suggesting that when the inter-class variance is large and the intra-class variance is relatively small, way fewer than maximum (1/5th of the data) PCA component capture most of the data variance. This will be evident in the “Scree plots”. The concordance of panel (**B**,**C**)’s cross-validation statistics suggests that the permuted model bearing mixed annotations in each class effectively reports the cross-validation statistics of a dataset that is equivalent to that of multiple sampling events from the mouse liver, an organ that is highly molecularly homogeneous and bears little intra-class variability in its 10 s PIRL-MS signatures. Note that in this example, less than 50 specimens per class provided reliable performance due to large inter-class and small intra-class variance in the mass spectral data.

The uncertainties discussed above, especially in the case of small studies or those with imbalanced datasets of divergent intra-class variance, necessitate rigorous secondary checks to ensure that the observed class distinctions are rooted in a “real” [biological] difference between datasets. Here, a powerful means to ensure that the observed class distinctions are not artifactual is provided through the creation of falsely annotated models (with mixed class representation), wherein each class is composed of an equal amount of data from all other participating classes. This model is subjected to the same number of principal components (PCs) and linear discriminant (LD) dimension components as the “true” model and should result in minimal or no class discrimination when subjected to supervised PCA-LDA modeling, as previously demonstrated [11,46]. This control model is valuable to present alongside the supervised training data model to ensure that class distinctions seen in a PCA-LDA study are unlikely to be caused by factors like artifacts of data overfitting or the incorrect selection of principal component numbers, among others. Likewise, the concordance of supervised and unsupervised clustering methods (such as PCA or k-means) and recovering and reporting of latent features seen in both methods are strong indications of the presence of “real” molecular differences between classes. This may not always be readily apparent, as LDA is designed to accentuate class differences in supervised analysis. This may create potential pitfalls in terms of data overfitting that must be mitigated. Figure 1A,B showcases the utility of a permutation test by using a mixed (false) class annotation as a suitable control. In a similar vein, a model created from data belonging to the same class being distributed equally across the same number of “pseudo-classes” should show poor distinction (Figure 1C). These are informative controls to perform to ensure that class distinctions that are seen are significant given the number of PCA components and data points per class utilized in the study.

### 3.3. Assessment of Molecular Model’s Robustness

**Figure 2:**
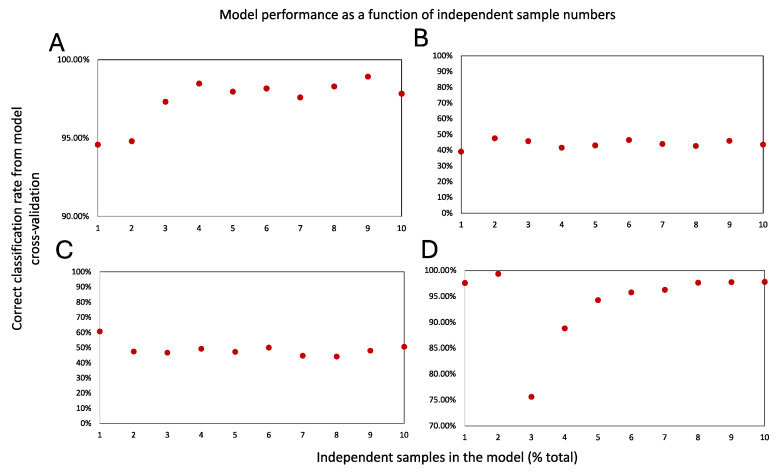
Learning curves created based on cross-validation statistics of Figure 1 PCA-LDA models. Cross-validation statistics of a series of PCA-LDA models created from various datasets with 10% increments of total data usage for Figure 1 models (where 1 denotes 10% data usage and 10 denotes 100%). These learning curves suggest that for models composed of highly molecularly distinct classes (such as Figure 1A), shown in panel (**A**), suitable cross-validation can be achieved at very low data utilization levels, past which the addition of further data fails to drastically impact the model’s predictive power. For permuted models that consistently sample the same heterogeneous class content across all data usage levels (Figure 1B), a poor(er) performance is seen, where in a similar vein, the addition of further data does not improve the performance (Panel (**B**)). To avoid overfitting, we adjusted the number of PCA components for each data usage accordingly. These values were 33 (for 10% data usage), 61, 89, 118, 151, 174, 198, 221, 239, and 258 (for 100% data usage) at each increment. In case the number of PCA components is not adjusted, for low data usage, overfitting takes place. Taking the learning curve of the Figure 1C model into consideration as a control for a highly homogeneous tissue-bearing low intra-class variability in panel (**C**), the learning curve from the permuted model of panel (**B**) appears to be similarly poorly performing. These learning curves were created from the same data that were utilized in Figure 1, as described previously [12]. Panel (**D**) shows the learning curve of Figure 1A, for which an inappropriately high number of PCA components of 258 across all data usages (except 10%, for which the maximum allowable was 165) was used. As can be seen here, overfitting takes place at low data usage following recovery and saturation at 80% of data usage.

A suitable molecular model must additionally possess favorable cross-validation statistics. A survey of the suitable cross-validation methods for various models is available and must be consulted [72]. In our experience, however, for large and balanced datasets (e.g., containing a similar number of data points in each class) that consequently require a large number of iterations to be suitable for this approach [73], and only in combination with appropriate controls such as permuted models (to address several inherent limitations [73]), a 20% leave-out cross-validation is a simple tool to assess a model’s robustness [11,12,46]. This test is a variant of the “leave *p*-out method”, wherein 20% of the data are iteratively removed and used to test the performance of the model that is built with the remaining 80% of the data. Here, one extreme scenario is the “leave-one-out” test, in which only one data point is removed and classified against the model, which is rebuilt iteratively with the rest of the data.This is more suitable for smaller datasets. While there have been calls for leave-out tests to be avoided altogether (especially in very small test sets [72,73]), they constitute an easy-to-implement strategy for first-pass model validation efforts if executed correctly (with cross-validation statistics reported alongside those of a similarly sized permuted model). Further, if the model is comprised of dependent data from the same specimen (e.g., multiple sampling events from the same specimen to capture heterogeneity), it is prudent to ensure that no data points from the same specimen populate both the training and test datasets to avoid bias [12,46]. Here, additionally, the creation of “learning curves” that plot the model’s performance metrics (e.g., cross-validation accuracy or sensitivity and specificity) as a function of independent specimen numbers that are used in the study (in increments of 10–20% total) will help glean further insights into whether the study sample size is sufficient or not [12,19]. A model that contains sufficient sample numbers should show a clear saturation of the “learning curves” upon utilization of the total study sample size [12]. Figure 2 displays the learning curves for the models that are shown in Figure 1. As can be seen here, the learning curve associated with Figure 1A (comprised of highly molecularly distinct classes) produces 94.58% correct classification from the cross-validation statistics, even at 10% of the total data usage (Figure 2A), and a plateau is reached past 40% of the total data usage. The permutated model containing mixed classes (Figure 2B), on the other hand, never reaches this level of performance metrics. Here, the inclusion of additional mixed-class data points does not improve the cross-validation performance of the model, which remains similar to the performance of the Figure 1C model comprising datapoints from the same specimens populating all participating classes (Figure 2C). For the learning curves in Figure 2A,B we adjusted the maximum number of PCA components based on one-fifth of the data at each usage interval. This was to avoid overfitting. Figure 2D shows the overfitting of the Figure 2A learning curve, in which we used a much larger maximum PC value at each interval (see legend). As can be seen here, at lower data usages of 10% and 20% the model data are severely overfitted, producing very high cross-validation values. This is followed with a drastic drop in the cross-validation statistics over the next few data usage intervals that eventually (as a suitable number of PC is attained) recovers to reach saturation. This is why it is critically important to evaluate the overfitting in multivariate models or move to validate the model’s generalizability using an independent validation set or with permutation tests, as suggested among others.

In addition to the leave-out approach discussed above, other methods of model validation could utilize a withheld dataset (e.g., 30% of data), followed by cross-validation of the classifier model (established using the 70% of training data) on the withheld data prior to further validating the classifier using an independent set of samples. An example is a study by Sans et al. [15], wherein the authors split the model data into training (using 70%) and test (using 30%) sets, as described above, and performed cross-validation, followed by subsequent classifier validation with an independent set of samples. We would like to additionally emphasize that many other cross-validation methods (e.g., *k*-fold and Montecarlo [72]) beyond the examples that we have used in our research exist, each with their own strengths and weaknesses, as summarized in [72], especially with respect to leave-out tests in small studies [73].

The model cross-validation efforts described above provide additional opportunities to access the so-called “confusion matrices” that detail the breakdown of model failures in terms of the classes that are involved. The confusion matrices are crucial to review to identify the classes that are most often “confused” with one another by the model. It may well be that despite the model’s failings, such class discrimination may not be crucial to the clinical case investigated. For example, it is possible that the course of treatment for the two classes that are indistinguishable by the model, often misclassified, happens to be similar, and as such, the need for their discrimination not so evident. In this case, these classes may be combined to enhance the model’s performance in the context of the clinical added value.

Lastly, the utility of the molecular model should be put to the test using an additional set of independent specimens, subjected to a classic training/test evaluation scheme. Here, we recommend that the MS operator and the data analyst be “blinded” to the ground truth class annotations of the test cohort, and that the models be tested using at least 50% of the sample sizes that were used to generate them. Mahalanobis distance calculations can be performed by subjecting the blind sample spectra to the same multivariate model, evaluated through overlays, as detailed above. The results of the cross-validation, the validation based on the internal withheld data subset, and the external validation with an independent sample set (as well as those of the resubstitution and permutation tests) must be presented using standard definitions of false positive and true negative rates in terms of the “sensitivity” and “specificity” of diagnosis [12,46]. At the very least, the results should be further interpreted using additional methods such as the area under receiver operating characteristic curve (AUROC) and Kappa statistics [29] to assess the robustness of the relationship between the data and the measured variables [74,75,76,77]. Any misclassified or outlier sample must be subjected to a detailed pathology assessment for elucidation of any aberrant histology or phenotype that is not accounted for in the model [11] and re-evaluated by further enriching the model to contain said missing elements for enhanced future utility [12]. Additionally, multisite validation and those by inexperienced users must also be performed as recommended [29] and implemented in cancer research [39].

For studies that use models that are generated from banked frozen tissue, we additionally recommend obtaining several prospectively acquired fresh tissue specimens to assess whether the presence of blood or body fluids can influence the modeling or prediction results, as recently examined [12,78]. Here, concordance between frozen and *in vivo* measurements of murine brain and fresh vs. frozen normal human skin has been established for PIRL-MS [12,78], but this observation may not scale to all cancer types and must be evaluated on a case-by-case basis. Here, a “sparse” multivariate analysis [11,12,46], excluding *m*/*z* features that are specific to the tissue origin or altered due to freezing/storage conditions (as opposed to using the entire resolvable *m*/*z* spectrum), can be used to glean insights into whether molecular models that are established from specimens of a given type or origin are sufficient to classify those from broader patient cohorts (e.g., can a model created from frozen banked tissue be used to classify prospectively acquired fresh surgical specimen?). While certain guidelines call for the model and test datasets to be composed of specimens from identical origins, biobank repositories often contain frozen tissue types from multiple sites and in numbers that may be challenging to acquire in a prospective study within a reasonable amount of time at a single centre. Here, it is worth mentioning that grouping the acquired data as a function of the specimen’s time in cold storage to monitor any potential spectral variance is highly recommended. Even if fresh and frozen tissues are shown to be comparable, however, we recommend adding as many banked specimens as possible to the model to assess whether a large population variance affects the plateau in the model’s “learning curves” discussed above, leading to a decrease in the “diagnostic power” due to variance-induced cluster overlap. This exercise can utilize the training and test data added together to boost the sample numbers.

In keeping with the recommendations provided above, Alewijn et al. [29] published a comprehensive summary of model building and validation efforts for multivariate analysis of adulterated food. The authors formalized various derived parameters beyond accuracy, sensitivity, and specificity as discussed above, including Kappa statistics [75,76], to ensure that the collected data accurately represent the measured variables. The authors also mentioned other methods such as AUROC and several probability-based methods that can potentially be used to evaluate a model’s performance, although no explicit endorsement of their use and/or limitations have been put forward. Consistent with our position, Alewijn et al. [29] also suggest challenges with the a priori calculation of specific sample numbers [29,30,79,80,81], hence buttressing our empirical approach of calculating “learning curves” as well as evaluating a model’s performance in the context of chance alone by way of randomly assigned class annotations. The proposed 20% [iterative] leave-out test above closely matches the recommendation of Alewijn et al. [29] for cross-validation and external validation, which is achieved by using the validation set, correlating the predictions to ground truth class annotations, ideally under blind conditions to remove any user bias. Additionally, Alewijn et al. [29] recommend a permutation test to evaluate overfitting (as a result of supervised analysis potentially accentuating class distinctions). In a permutation test, class assignments are randomly distributed between the model data points. Here, our mixed class (false) annotation proposal essentially captures the same analysis. Finally, consistent with the repeatability test on replicate measurements that is performed from each independent specimen [29], we suggest multiple measurements on the same specimen and calculating the concordance for spatially invariant correct class assignments. While there are parallels between the approach taken by our group and the recommendations made by Alewijn et al. [29], we strongly endorse their publication and encourage users to closely consult the recommendations published therein. Of particular interest is their proposal of model cross-validation probability distribution (together with external probability distribution), which must utilize a reasonably balanced dataset, aiming to assess the capability of the model when confronted with “new” samples (first faced with samples in the training set and subsequently with an independent batch). Consistent with their proposals, we also support expanded validation using large datasets in a multisite manner, including by inexperienced users. Here, we would like to draw attention to other strategies in data modeling and interpretation that may specifically address a model’s generalizability (i.e., its performance when encountering new or noisy data or those whose influence is not captured in the model). Our current strategies have largely focused on including as much diversity in the model as possible in the hopes to also improve its generalizability. This may come at the cost of reduced cross-validation, and we thus encourage readers to pursue other proposed strategies to boost generalizability, which may be independent of attempts to capture the essence of new data in it by incorporating diversity [82].

## 4. Interpretation of Data

### 4.1. The Need to Understand the Classifiers

**Figure 3:**
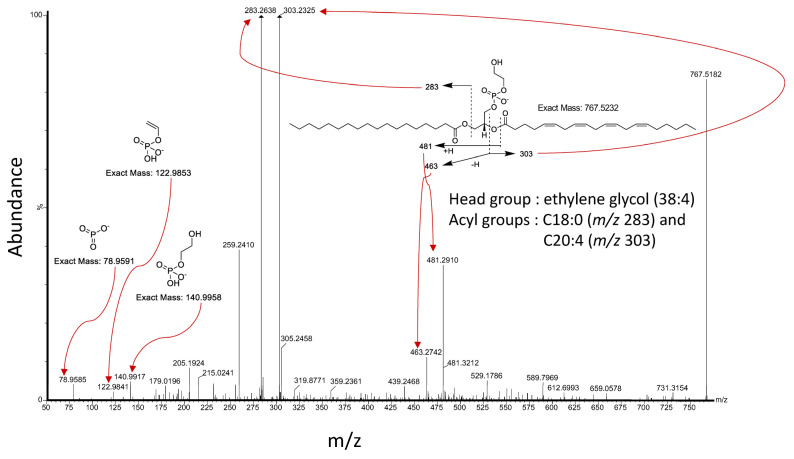
An unusual lipid with ethylene glycol headgroup seen in PIRL-MS analysis of skin cancer. This previously unreported lipid was seen in our attempt to discover biomarker ions for skin cancer type differentiation with PIRL-MS [12]. In this figure, we are showing the MS/MS spectrum of the parent ion *m*/*z* 767.5232, obtained from extracts made from the plume of PIRL laser collected from cancer tissue by capture on a filter paper, as described in [12], and subsequently analyzed with ElectroSpray Ionization (ESI) with high-resolution mass spectrometry [12] post-chromatography. Surprisingly, this ion was not seen in lipid extracts made directly from the tissue, which may further imply a role for the desorption source in its formation (pending future additional clarifying experiments to be performed). This unusual headgroup is not reported in LipidMaps [97], which is routinely used by us and many other groups to verify tentative identity assignments for lipidic species.

The supervised approaches for the modeling of mass spectral data described above largely utilize most or all the resolvable *m*/*z* features, and as such, they remain vulnerable to the influence of artifactual contributions to their mass spectral profiles. While multivariate analyses inherently boil down to utilizing a feature list in the classifier (reduced list of most discriminating *m*/*z* features), it is important that all the *m*/*z* values that have been shown to be important for class distinctions be identified and demonstrated to “make [biological] sense”. These *m*/*z* values can be selected (for downstream identification) by way of analyzing the so-called “loading plots”, which demonstrate the rank order of each *m*/*z* value for its strength of contribution to the overall class discrimination. For example, in a hypothetical case, the artifactual discrimination between healthy and diseased tissue could be driven by metabolic products of an exogenous therapeutic agent taken by the patient before biobanking, as opposed to true biological differences in tissue chemistry driven by the disease. Therefore, the identity of tissue-type-determining *m/z* values must be established to rule out any artifactual discriminations being made inadvertently by the algorithm that is used to drive class distinctions. In a previous study to discover biomarker ions for 10 s skin cancer type differentiation with PIRL-MS, we encountered an unusual lipid (*m/z* 767.5232) with a previously unreported ethylene glycol (38:4) headgroup and C18:0 and C20:4 acyl groups, tentatively described as contributing strongly to class discrimination (Figure 3). As the presence of ethylene glycol (despite its wide use outside the laboratory [83] and in some experimental lipid-based encapsulating therapeutic agents [84,85]) could not be rationalized in the context of patient-derived specimens, we excluded this ion as a classifier for skin cancer type differentiation from our published report [12]. Here, it must be emphasized that excluding ions solely because of their “unusual” nature may not always be warranted. Further investigations as to the origin of such unusual ions must be conducted. If these ions are shown to be biologically relevant, they will add a wealth of information regarding metabolic pathways. On the other hand, merely detecting a previously unreported ion in a biological specimen does not warrant de facto considering such ion a putative marker without carefully evaluating the experimental design and conditions to rule out artifactual inclusions. In a similar vein, the same concerns have existed in the untargeted mass spectrometry analysis of food products involving oleamide. This compound is an endogenous metabolite involved in the sleep cycle [86] and happens to also be a common plastic additive [87,88] that is known to leach into specimens stored in plastic containers [89,90]. Oleamide has been reported in honey [91] and in herbal supplement QC studies [92], even taking the role of an [accidental] adulterant in several reports. Interestingly, the same ubiquitous oleamide compound has also been observed in untargeted urinary analysis of bladder cancer patients [93], laryngeal cancer patients [94], and renal cancer patients [95], as well as in the serum of patients with colorectal cancer [93,96]. In addition, due to the widespread use of oleamide-containing plasticizers in everyday products, this compound has also been steadily introduced into the human body over the past several years [90]. This creates a need for a careful reconsideration of both specimen storage container types and patient oral intake history (if feasible) to ensure the validity of conclusions made from oleamide detection in studies involving human subjects or specimens. These observations highlight an important failing with respect to a sole reliance on statistical associations for biomarker discovery and justify the need for elucidation of molecular identities and parallel investigations to rationalize their involvement in class differentiations. For example, an identified metabolite that is known to be involved in a disease pathway through genomic studies is much more robustly implicated as a true disease marker than an ion without parallel evidence buttressing its involvement in said disease pathway. Here, a tentative assignment of ion identities should constitute the bare minimum to at least rule out said artifacts, as discussed above. However, as genomes and phenomes do not always exhibit a clear one-to-one correspondence, we do not intend this recommendation to mean that an analyte which is robustly identified yet cannot be rationalized by genomic changes is less rigorously implicated in the disease biology.

### 4.2. Revising the Model Based on Rationalizable Classifiers and Further Validating It

Once the identities of the most important *m/z* features (e.g., ions in this case) contributing to the strengths of the statistical discrimination are established, re-evaluating and rebuilding multivariate models using feature-based analysis of known, identified, and biologically relevant classifier ions via a “sparse [*m/z*] analysis” must be carried out. This requires unequivocally establishing ion identities for tens of *m*/*z* features, revealed by the “loading plot” that is implicated most strongly in class distinctions (similarly, also by shrinkage in LASSO or recursive features elimination, as two examples [98]). As most hand-held ambient MS analysis probes often do not require highly accurate elucidation of molecular masses to provide class distinctions, they are frequently used in tandem with “medium”-resolution mass analyzers. This creates a problem for the accurate identification of analytes with MS^1^ or MS^2^ approaches in real time, especially in the absence of chromatographic separation. To make the matter more complicated, many ambient MS probes create transient and weak signals that are essentially unsuitable for online MS^2^ approaches on most mass analyzers. Here, while utilizing high-resolution mass analyzers and ion trap technologies may address both the inherent limitations of sensitivity and transient signal accumulation, a lack of chromatographic separation remains an issue to contend with. We have trapped the laser ablation plume on a filter paper and compared the chromatography retention profiles of said laser plume to that of the conventional lipid extract prepared directly from the tissue, subjected to the same extraction and ionization method as the plume-containing filter paper, showing general concordance between the retention profiles in an UPLC-MS^2^ experiment [11]. However, the use of very different desorption methods (laser-based in PIRL and electrospray-based in UPLC-MS^2^) is bound to result in some differences in the obtained spectral profiles. Having said this, barring the uncertainty associated with matching *m*/*z* peaks that are informed by loading plot analysis of online PIRL-MS profiles (on a Waters Xevo G2XS time-of-flight instrument with resolving power of ~20,000 FWHM) to that of the UPLC-MS^1^ runs (on a Waters Synapt G2-S with the resolving power of ~100,000 FWHM) utilizing the most abundant peaks from the same spectral bins in both experiments, this combination has allowed us to use the accurate mass and MS^2^ analysis after chromatography to determine ion assignments for tens of top PIRL-MS-classifying *m/z* values across many human cancer class distinctions utilizing chromatographic separation with UPLC-MS^2^ analysis [11,12,46]. In these studies, starting from a list of the top 100 most class-identifying *m/z* values, we have often been able to assign identities to between 20 and 30 ions. Due to experimental design, not all ions that were informed by online analysis of the laser plume have been observed in the tissue (or captured laser plume) extract in sufficient amounts for MS^2^ analysis, and this has been the most common source of failure. Nevertheless, this list of 20–30 ions has been successful in maintaining good model performance in a feature-based (or “sparse”) analysis, wherein class (or cluster) distances are calculated only based on the identified *m/z* feature list. These feature-based models have further shown success in high-sensitivity and -specificity prediction of correct pathological classes for the blind specimens through sparse analysis [12,46]. Through this scheme, we have been able to at least avoid artifactual class distinctions based on *m/z* features that are not known to be biologically relevant (i.e., all hits were found to be associated with native human tissue in the LipidMaps [97] database, devoid of therapeutic by-products or tissue storage artifacts such as embedding or antifreeze monomers). Here, it must be emphasized that coupling PIRL-MS with a high-resolution ion trap instrument to address signal accumulation for reliable MS^2^ (as opposed to a transient time-of-flight) appears to be more pressing than the use of chromatography. The concordance of multivariate models built with 100 mDa to 1 Da bins speaks to a muted influence of overlapping peaks (separable in chromatography), at least for the ion source that is utilized in PIRL-MS. However, online analysis using a high-resolution analyzer is required to verify this hypothesis and to resolve true overlaps that are invisible to us in our current setup due to its limited resolving power, as discussed above. The availability of ion identities for model classifiers serves many purposes: (1) it shields against false discoveries based on non-biological contaminants that could otherwise serve as strong classifiers (e.g., accidental contamination with embedding material due to the alteration of the banking process or taking a pharmacological supplement or therapeutics), (2) it allows for the rational refinement of molecular models for concordant classification of fresh vs. frozen tissue, (3) it further adds molecular-level details to disease metabolic pathways, and (4) it provides an opportunity to rationally evaluate the inclusion/exclusion of classifying *m*/*z* features based on a parallel understanding of disease pathways and the “noise” level of said feature at the population level to work towards improving model robustness.

**Figure 4:**
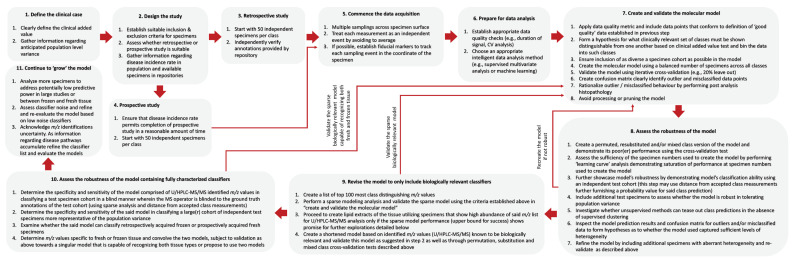
The workflow for creation, evaluation, and refinement of molecular models for biological tissue type classification with untargeted mass spectrometry. Here, we have summarized our vision for how molecular models can best be created evaluated and refined towards rigorous use in future clinical decision-making (additional details are provided in the text regarding each step). While the provided pictorial summary largely captures the lessons learned throughout PIRL-MS developments in our group, the principles that are summarized in this figure may be relevant to other untargeted MS analysis approaches used in parallel or closely related studies.

Summarizing the discussions above, Figure 4 defines the workflow for a robust design and meaningful interpretation of PIRL-MS modeling results that we deem to be relevant to investigators utilizing similar technologies for the rapid identification of biological tissues with untargeted mass spectrometry. We envision the molecular models created and validated using the guidelines that are summarized in Figure 4 to form “living documents” that require continual updates and refinement as additional knowledge regarding disease molecular pathways or tissue molecular heterogeneity at the population level surfaces, necessitating adaptations such as shortening the classifier list or adapting the standard deviation level for distance-dependent [from a cluster] classification methods. While this strategy leads to inevitable recertification of the new models by regulatory bodies, we believe that the potential improvements gained in diagnostic accuracy of such revised models may well justify the effort.

## 5. Caveats and Additional Points to Consider

We acknowledge several caveats and technical uncertainties associated with confidence in determination of molecular identities using the current implementation of hardware that is available to us. Namely, the hindering resolution of the mass analyzer used, the absence of on-line MS^2^ performed directly on laser-extracted ions, and the reliance on *m*/*z* matching across laser-extracted (for PIRL-MS) and solvent-extracted (for UPLC-MS^2^ with ESI) techniques performed on different instruments. We have additionally left out many nuances concerning the specifics of the hardware operation, which are deemed to be of little interest to the broad readership of this manuscript. To this end, however, we encourage readers to establish their own standard operating procedures (SOPs) utilizing the high-level points discussed in this manuscript and beyond, capturing pitfalls of implementation using their observations of hardware performance, and taking the necessary steps to guard against false discoveries. Some implementations may suffer from (e.g.,) signal carryover or require an assessment of carryover or signal contamination. Such important points must be taken into consideration in data quality checks and were not discussed herein in the context of our observations with PIRL-MS. Likewise, we did not discuss various methods of intelligent data analysis that are reported to be applicable to untargeted analysis with mass spectrometry. We encourage readers to closely monitor recent advances in data analytics, especially with respect to data-driven or hierarchical methods that are suitable for mass spectrometry datasets. Lastly, it must be emphasized that experimental conditions as well as data analysis methods, parameters, and considerations discussed throughout this manuscript must be carefully documented and reported. To this end, efforts have been directed at creating guidelines for documenting and reporting key variables in untargeted analysis. These guidelines (as part of the standardization of reporting metrics) have been provided by Peter et al. [99], and we encourage users to consult this manuscript, as well as the related web platform (https://nontargetedanalysis.org, 13 March 2024). Through the proposed non-targeted analysis (NTA) Study Reporting Tool (SRT) therein, investigators will be able to accurately (1) identify, (2) optimize, (3) document, and (4) report key parameters related to study design, experimental implementation, data quality, data analysis, and necessary controls. Through adhering to these guidelines, an added benefit of increased transparency in the dissemination of scientific results will be achieved. This will facilitate verification and study reproducibility assessment efforts. Insufficient reporting of methodological parameters (especially those with a significant impact on study results) can hamper the rigorous assessment of the quality of the disseminated knowledge, in the absence of which the validity of the conclusions that are drawn and reported could not be established independently. While it is universally accepted that a carefully presented scientific report should contain sufficient details to enable those who are skilled in the art to replicate the study findings, the current trends in the peer-review process rarely subject methodological details to in-depth scrutiny, especially with respect to the comprehensiveness of the information included. In this vein, efforts by Peter et al. [99] to create guidelines for improved comprehensiveness and transparency of methodological details in untargeted analysis reports are both invaluable and much needed. Lastly, it must be emphasized that in this manuscript, we have not touched upon the pros and cons of various statistical analysis methods that are suitable for mass spectrometry research. In addition, we have not provided sufficient guidance for users to plan their next data analysis steps, in case the chosen method fails to produce results (e.g., when the permutation test for one method fails). Addressing these issues requires consultation with a statistician in the study design. Likewise, approaches based on handheld MS probes for clinical diagnosis have largely involved linear methods, which are more suitable for smaller datasets [50]. As the number of datasets collected by hand-held MS analysis probes increases, so does their appeal for non-linear methods such as neuronal networks, among others. We strongly recommend readers to stay up-to-date with respect to such methodological developments, especially in the field of mass spectrometry imaging, which has been producing large(r) datasets for a number of years and is “ahead” of hand-held methods in terms of data analysis [100]. Here, it must be emphasized that the chosen method should not be computationally extensive, so as to not hamper the goal of being able to deliver robust clinical diagnosis in a short period of time, which is the focus of our manuscript here.
